# Body Fat Depletion: the Yin Paradigm for Treating Type 2 Diabetes

**DOI:** 10.1007/s11883-023-01181-4

**Published:** 2023-12-27

**Authors:** Jingjing Zhu, John P. H. Wilding

**Affiliations:** 1 Department of Endocrinology and Metabolism, the Second Affiliated Hospital of Soochow University, Suzhou, Jiangsu People’s Republic of China; 2 Department of Cardiovascular and Metabolic Medicine, Institute of Life Course and Medical Sciences, University of Liverpool, Liverpool, UK; 3 Clinical Sciences Centre, Liverpool University Hospitals NHS Foundation Trust, Longmoor Lane, Liverpool, UK

**Keywords:** Type 2 diabetes, Body fat, Sodium-glucose cotransporter-2 inhibitor, Incretin, Metabolic benefit

## Abstract

**Purpose of Review:**

To highlight that body fat depletion (the Yin paradigm) with glucose-lowering treatments (the Yang paradigm) are associated with metabolic benefits for patients with type 2 diabetes mellitus (T2DM).

**Recent Findings:**

The sodium-glucose cotransporter-2 inhibitor-mediated sodium/glucose deprivation can directly improve glycemic control and kidney outcome in patients with T2DM. The glucose deprivation might also promote systemic fatty acid β-oxidation to deplete ectopic/visceral fat and thereby contribute to the prevention of cardiovascular diseases. As with metabolic surgery, bioengineered incretin-based medications with potent anorexigenic and insulinotropic efficacy can significantly reduce blood glucose as well as body weight (especially in the ectopic/visceral fat depots). The latter effects could be a key contributor to their cardiovascular-renal protective effects.

**Summary:**

In addition to a healthy diet, the newer glucose-lowering medications, with body fat reduction effects, should be prioritized when treating patients with T2DM, especially for those with established cardiovascular/renal risks or diseases.

## Introduction

A body of evidence indicates that excessive adiposity, especially in the visceral (adipose tissue) and ectopic (non-adipose tissue) sites, are strongly associated with an increased risk of developing type 2 diabetes mellitus (T2DM) [1] and its various complications [2, 3]. It has long been recognized that metabolic dysfunction-associated steatotic liver disease (MASLD) is a definitive risk factor for the development and progression of T2DM, whereas improvement/resolution of MASLD can significantly reduce the risk [4]. The liver plays a critical role in maintaining glucose homeostasis. Immune cell infiltration occurs concurrently as ectopic fat, i.e., triacylglycerols (TG) accumulates in hepatocytes [5]. This state of chronic inflammation can suppress peroxisome proliferator-activated receptor γ (PPARγ) and impair the conversion of fatty acids (FA) to TG [6], which could result in excess intracellular FA blocking insulin signaling pathways to potentiate hepatic insulin resistance [7, 8]. Secondary to pancreatic fat accumulation, excess intracellular FA could likewise interrupt signaling pathways involved in glucose sensing and insulin secretion [9]. This is consistent with observations that pancreatic fat content is positively correlated with glucose intolerance; patients with T2DM have higher amounts of pancreatic fat compared with those without diabetes [10]. Hypothalamic fat accumulation accompanied with chronic inflammation could also occur in T2DM and obesity [11, 12]. As plasma FA levels rise before meals and fall with feeding/glucose influx [13], hypothalamic FA might serve as another strategic intracellular signal in regulating energy homeostasis, i.e., stimulating appetite when plasma/hypothalamic FA is high and promoting satiety once the level falls [14]. This state of chronic inflammation might also lead to excess intracellular FA favoring appetite over satiety and thereby aggravate adiposity and contribute to the adverse metabolic outcomes [15]. Therefore, this “ectopic fat accumulation → (chronic inflammation-mediated PPARγ suppression) → excess intracellular FA elicited-dysfunction” might be pivotal to the development and progression of T2DM.

Besides accumulating ectopic fat, the heart and kidneys (where micro/macrovascular complications of T2DM mostly occur [16, 17]) also possess “genuine” adipose tissue (e.g., epi/pericardial fat and peri/intra renal fat, which is classified into visceral adipose tissue) [18]. Clinical studies have confirmed that patients with T2DM have relatively higher proportions of epicardial, pericardial, and renal fat [19, 20]. Excess FA released from these visceral depots may enter adjacent cells/structures such as cardiomyocytes (including the conduction system), renal tubules, and coronary/renal endothelial cells, disrupt multiple cellular signaling, and elicit metabolic (dysfunction)-associated organ damage [21] including cardiomyopathy, arrhythmias (e.g., atrial fibrillation), coronary artery diseases [22], hypertension, and chronic kidney disease [20].

**Fig. 1:**
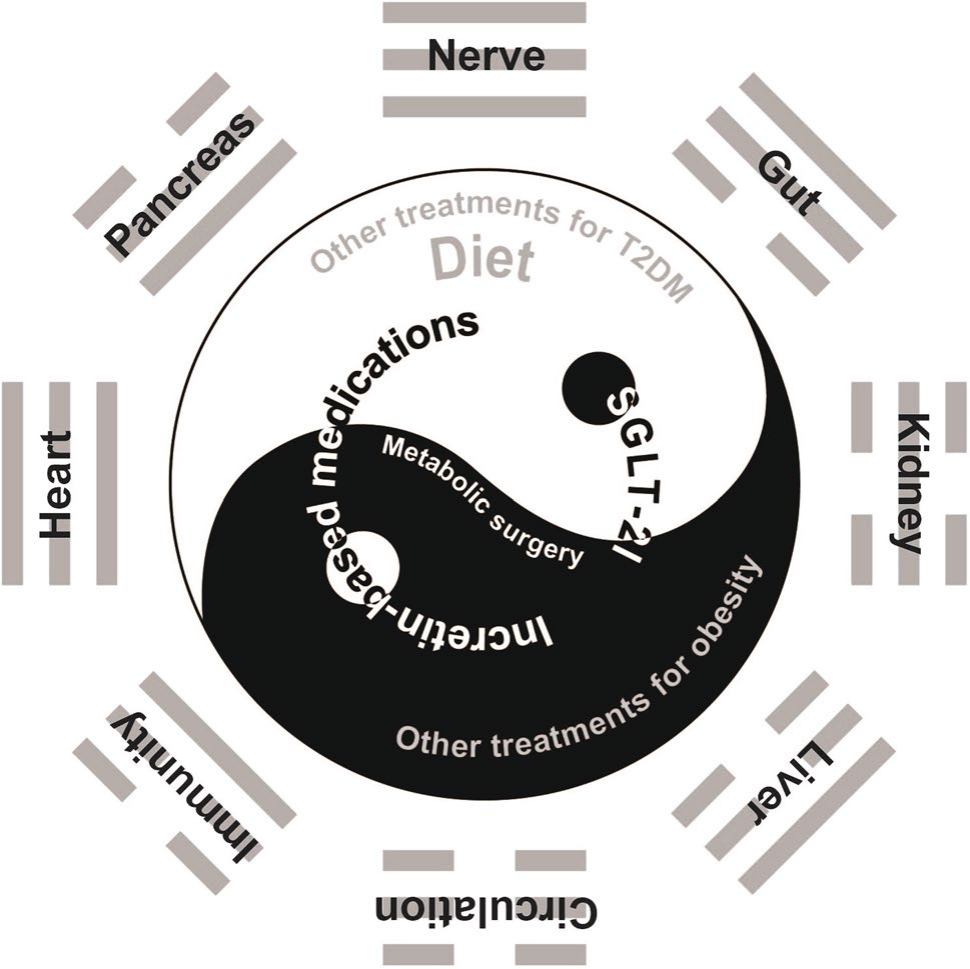
The Yin and Yang paradigms for treating type 2 diabetes and improving metabolic outcomes

It is worth noting that targeting the downstream effect of ectopic/visceral fat accumulation, i.e., chronic/meta-inflammation, might favor PPARγ-associated adiposity and bring no apparent metabolic benefits to patients with T2DM [23]. Therefore, if the Yang paradigm for treating T2DM focused on lowering blood glucose (treatments in the white background or denoted in white), the Yin paradigm—body fat depletion (by way of body weight reduction) (treatments in the black background or denoted in black), must never be neglected (Fig. 1). Weight management has always been designated as one of the most important components in primary care for T2DM. The *Standards of Care in Diabetes-2023* (*SCD2023*) published by the American Diabetes Association recommends that most people with T2DM are supported with lifestyle modifications (including nutrition, physical activity, and behavioral therapy) with the aim of achieving and maintaining ≥ 5% weight loss; the goal can be set higher (up to 15%) when newer glucose-lowering medications with body weight reduction effects are included in the treatment regimen [24].

In this review, glucose-lowering treatments with body weight reduction effects are broadly categorized into non-pharmaceutical and pharmaceutical approaches. We essentially summarize their glucose-lowering efficacies and contemplate on how they can deplete body fat to determine the metabolic outcomes of patients with T2DM.

## Diet

Diet has always been an important component in the treatment of T2DM. A recently published systematic review and meta-analysis of randomized controlled trials (RCT) [25] shows that compared with usual diet, energy-restricted diet increased rate of T2DM remission (hemoglobin A1c (HbA1c) < 6.5% with no antidiabetic medication) at a 2-year follow-up by 10 more per 100 patients (data from the LOOK AHEAD study [26] and the DiRECT [27, 28] were both included). The dose–response analysis indicates that at 6 months, each 500 kcal/day decrease in energy intake resulted in clinically meaningful reductions in HbA1c (MD (mean difference) =  − 0.82%). However, the glucose-lowering efficacy attenuated as the intervention progressed and became negligible after 12 months [25]. Though not energy-restricted, (compared with control (e.g., low-fat diet), low (< 130 g/day), or very low (< 50 g/day) carbohydrate/ketogenic diet could slightly increase T2DM remission rate (by 5 more per 100 patients at a 6-month follow-up); achieve a higher reduction in HbA1c (MD =  − 0.47% and − 0.23% at 6 and 12 months) [29]. However, the safety of a low-carbohydrate or very low-carbohydrate diet is still a major concern, especially for patients with relative insulin deficiency (rather than insulin resistance/hyperinsulinemia) in T2DM [30].

Energy-restricted diet has limited efficacy in reducing body fat. The LOOK AHEAD study demonstrates that across an 8-year time interval, the difference of weight reduction between the control and intensive lifestyle intervention (i.e., low-energy diet combined with increased physical exercise) groups was only modest as evidenced by − 3.5% (of the baseline body weight) vs. − 6.0% [31]; the ratio of reduction in fat to lean body mass was approximately 2 in the intervention group, weight regain in this group was nearly 100% fat body mass [32]. This may partly explain why the intensive lifestyle intervention was not associated with lower risks for major adverse cardiovascular events (MACE) defined as nonfatal stroke, nonfatal myocardial infarction, and cardiovascular death) or other cardiovascular diseases including hospitalization for heart failure or angina and atrial fibrillation in patients with T2DM [33, 34]. Further evidence indicates that maintainers (in both groups) who kept off ≥ 75% of weight loss achieved the greatest cardiovascular benefit [35]. A series of post hoc analyses also suggest that there could be a negative correlation between the magnitude of weight reduction (especially in visceral fat depot) and the incidence of cardiovascular disease (especially for heart failure) [36–38]. Therefore, body weight/fat reduction is the key in improving the cardiovascular outcomes in patients with T2DM. Dose–response analysis of body fat reduction and prevention of adverse metabolic events might be needed to further understand these effects. Besides body composition, whether the dynamics of FA could determine cardiovascular risks for patients with T2DM is another interesting research area to be explored [39].

## Metabolic Surgery

Metabolic/bariatric surgery is not only the most effective approach for weight reduction but obesity-related comorbidities and quality of life can also be significantly improved after surgery [40]. The *SCD2023* recommends that metabolic surgery should be considered to treat T2DM in adult patients with class III obesity (categorized according to body mass index (BMI) and ethnicity) or class II obesity who receive nonsurgical treatment but do not achieve durable weight reduction and improvement in comorbidities [24]. Compared with nonsurgical treatment, metabolic surgery was associated with a greater reduction in HbA1c (MD =  − 3.1% at endpoint) and rates of T2DM remission (odds ratio (OR) = 4.23) [41].

A body composition study showed that the depletion ratio of fat to lean body mass was 3.3 in (baseline BMI = 43.4 kg/m^2^) patients with total weight reduction < 50% after metabolic surgery; this ratio could rise to 5.3 in (baseline BMI = 41.4 kg/m^2^) patients achieved higher weight reduction (≥ 50%) [42]. Long-term follow-up (up to 14 years) indicates in (baseline BMI = 46.6 kg/m^2^) patients receiving metabolic surgery liver fat was significantly reduced as evidenced by a 9% increase in liver-to-spleen density; there can be a positive correlation between liver fat reduction and incidence of post-surgical T2DM remission [43]. Estimated proportions of fat depletion in subcutaneous, visceral, and epicardial depots after metabolic surgery were 20%, 42%, and 30% [43]. This extensive body fat depletion, especially in the ectopic and visceral depots, may contribute to the prevention of major complications of T2DM [43]. Observational studies suggest that MACE was significantly lower (OR = 0.49) in patients with T2DM receiving metabolic surgery [44]. Risks for heart failure and atrial fibrillation may also be reduced, though the results were less conclusive [44]. Furthermore, preclinical and clinical studies suggest that metabolic surgery may prevent the progression of chronic kidney disease and improve renal outcomes in T2DM [45].

Metabolic surgery might remodel neural signaling in the gut-brain axis to exaggerate postprandial insulin secretion (under a relatively inadequate food/glucose stimulation) [46, 47]. In some patients, this is excessive and leads to hyperinsulinemic hypoglycemia syndrome after Roux-en-Y gastric bypass [47]. The exaggerated (portal) insulin secretion could not only improve glycemic control but also increase very low-density lipoprotein (VLDL) output to deplete liver TG/fat [43, 48]. Theoretically, lipoprotein (LPL) affinity for VLDL-TG hydrolysis is lower in subcutaneous than visceral depots; hence, the increased VLDL-TG output might also shift fat distribution from visceral to subcutaneous depot and thereby decrease the ratio of visceral to subcutaneous fat volume (R) [49]. The post-surgical R could be calculated as $\frac{\mathrm{presurgical visceral fat volume}/1 -\mathrm{ postsurgical visceral fat depletion}/2}{\mathrm{presurgical subcutaneous fat volume}/3 -\mathrm{ postsurgical subcutaneous fat depletion}/4}$. As 1 and 3 and the sum of 2 and 4 are constant in a bariatric surgery patient, when the R decreases, the proportion of fat depletion in the visceral depot (*i.e.*, 2/1) will, on the contrary, increase. Taken together, compared with “negative energy balance matched with decreased insulin secretion” i.e., diet, it might be this discrepancy that “negative energy balance matched with increased insulin secretion and VLDL output” makes metabolic surgery much more efficient in visceral/ectopic fat depletion and (hence) beneficial in T2DM remission and prevention of cardiovascular/renal complications. Further kinetic models of the substrate (VLDL-TG) competition between subcutaneous and visceral LPL are needed to confirm this hypothesis.

## Pharmacology

Glycemic control with some particular classes of antidiabetic medications including thiazolidinediones (TZD), sulfonylureas, and insulin analogs is at the expense of body fat gain [50]. Although there has been some evidence that pioglitazone could significantly reduce MACE in patients with T2DM [51], this is not the case for all TZD, especially for rosiglitazone, which increased risk for myocardial infarction but not for cardiovascular or all-cause mortality [52]. Moreover, TZD are generally associated with a high risk for the development of congestive heart failure [53]. These unfavorable cardiovascular outcomes might be attributed to the TZD-induced predominant subcutaneous (rather than visceral) fat accumulation [54]. As a consequence, excess subcutaneous fat/FA can likewise elicit systemic endothelial dysfunction, injure the peripheral nervous system, and increase risks for developing/deteriorating macro- and microvascular complications [55, 56]. Therefore, those newer glucose-lowering medications with weight reduction effects have become the most recommended second-line and, in some cases, first-line antidiabetic treatment [57].

## Sodium-Glucose Cotransporter-2 Inhibitor (SGLT-2I)

The SGLT-2I blocks glucose and sodium reabsorption in the renal proximal tubules and thereby promotes their excretion into urine [58], hence this is the only class of glucose-lowering medications that “genuinely” deplete (rather than relocate) blood glucose. A meta-analysis of 66 RCT indicated that the glucose-lowering efficacy of SGLT-2I was persistent with significant HbA1c reduction at week 12 (MD =  − 0.63%), 24 (MD =  − 0.63%), 52 (MD =  − 0.66%), and 104 (MD =  − 0.60%) (compared with either placebo/other antidiabetic treatments) [59]. Major cardiovascular outcome trials (CVOT) of SGLT-2I suggest that the glucose-lowering efficacy is maintained (MD =  − 0.36 to − 0.58%) for at least 3 to 4 years [60–62].

The SGLT-2I-mediated glucose deprivation could reduce body weight/fat [58, 63]. Clinical evidence demonstrated that the SGLT-2I could exert progressive BMI reduction at 12 (MD =  − 0.52 kg/m^2^), 24 (MD =  − 0.73 kg/m^2^), 52 (− 0.93 kg/m^2^), and 104 (− 1.22 kg/m^2^) weeks [59]. Further body composition studies showed that within the duration of treatment, lean body mass remained stable [64], while fat depletion in subcutaneous, visceral, and liver/ectopic sites were all significant [65]. Specifically, the SGLT-2I could deplete liver fat via glucose deprivation (as de novo lipogenesis accounts for one-fourth of hepatic FA content in MASLD) [66] and glucose deprivation-boosted hepatic FA β-oxidation. The improved hepatic insulin sensitivity could be another important contributor to the persistent glucose-lowering efficacy of SGLT-2I.

The *SCD2023* recommends that a SGLT-2I should be included in the treatment regimen for patients with T2DM and established cardiovascular/renal disease or risks [67], as the major CVOTs indicated that risks of MACE (HR = 0.89) and cardiovascular death or hospitalization for heart failure (HR = 0.77) were all significantly reduced in patients with T2DM receiving SGLT-2I treatment [68••], though the SGLT-2I is associated with a statistically but not clinically significant increase in total plasma cholesterol (MD = 0.003 mmol/L) [69] and an insignificant depletion in epicardial fat [65]. Moreover, a pooled analysis of 31 RCT (including all up-to-date CVOT) demonstrated that compared with placebo or no therapy, the SGLT-2I could also significantly reduce the incidence of total (risk ratio (RR) = 0.83) and serious (RR = 0.75) atrial fibrillation [70]. To achieve such extensive cardiovascular benefits, in addition to reducing plasma glucose, intravascular volume, and weight, the SGLT-2I may specifically boost FA β-oxidation in cardiomyocytes (for they have rather high energy demands) to alleviate myocardial lipotoxicity [71]. The improved myocardial (including conduction) function may in turn initiate metabolic reprogramming in the cardiac microenvironment and thereby correct coronary endothelial dysfunction [72].

The SGLT-2I is also considered as nephroprotective, since it can increase sodium delivery to the distal tubules, inhibit tubuloglomerular feedback to constrict the afferent arteriole, and reduce intraglomerular pressure [73]. Major renal outcome trials (with median follow-up ranging from 1.3 to 2.6 years) confirmed that the SGLT-2I significantly improved the composite renal outcomes (including worsening of renal function, end-stage renal disease and renal death) with a hazard ratio (HR) of 0.60 [74••]. Similarly, real-world evidence (with a mean follow-up of 1.2 years) demonstrated that (compared with other antidiabetic medications) initiation of the SGLT-2I was associated with reduced risk of composite renal event (HR = 0.49) [75].

## Incretin-Based Medications

### Glucagon-Like Peptide 1 Receptor Agonist (GLP-1RA)

GLP-1 is a glucose-dependent insulinotropic hormone secreted from intestinal L cells [76]. A body of evidence suggests that GLP-1 is capable of preventing adverse metabolic outcomes via regulating gastrointestinal, neuromuscular, endocrine (including adipose tissue), and even immune systems [76]. Considering the short half-life (only 2 min) of endogenous GLP-1 (due to dipeptidyl peptidase 4 (DPP-4) degradation), a range of DPP-4 resistant-GLP-1RA (with extended half-life), most of which are in subcutaneous (sc) rather than oral (po) peptide formulation, have been developed for the treatment of metabolic disorders including T2DM and obesity [77]. Major CVOT (with a mean follow-up of 2.1 and 5.4 years) demonstrated that compared with placebo, long-acting GLP-1A—dulaglutide (1.5 mg qw sc) and semaglutide (1.0 mg qw sc) significantly reduced HbA1c (MD =  − 0.60%, − 1.40%) in patients with T2DM (compared with placebo) [78, 79]. The *SCD2023* recommends that a GLP-1RA is preferred to insulin (when possible) for patients with T2DM [67]. A meta-analysis of 18 RCTs (with a mean follow-up of 16 to 58 weeks) further confirmed that compared with basal insulin, the long-acting GLP-1RA were associated with a greater reduction in HbA1c (MD =  − 0.27%), whereas the short-acting GLP-1RA (exenatide 10 μg bid sc) did not perform significantly better [80].

With respect to the anorexigenic effect of the GLP-1RA [76], data from the major CVOT indicated that long-acting GLP-1RA—liraglutide (1.8 mg qd sc) and semaglutide (1.0 mg qw sc) were associated with 3% and 5% of body weight reduction from baseline (BMI = 32.5 and 32.9 kg/m^2^) in patients receiving the treatment for 3.5 and 2.1 years, respectively [79, 81]. Higher doses of liraglutide (3 mg qd sc) and semaglutide (2.4 mg qd sc) have been approved for the treatment of obesity [82, 83]. Moreover, a recent published phase 3 RCT suggests that semaglutide (50 mg qd po) is as effective as semaglutide (2.4 mg qw sc) in reducing body weight among patients with overweight or obesity [84]. As part of the clinical development, these doses have also been tested in patients with T2DM with higher BMI (37.0 and 35.7 kg/m^2^) and resulted in reduction of 6% and 10% of body weight from baseline (vs. 4.7% for liraglutide 1.8 mg and 7% for semaglutide 1 mg) in 56 and 68 weeks, respectively [85, 86]. Taken together, there seem to be positive correlations between baseline BMI/dose of GLP-1RA and body weight reduction in patients with T2DM receiving GLP-1RA treatment. Further body composition studies demonstrated that for patients with overweight or obesity (BMI = 37.8 kg/m^2^) receiving semaglutide (2.4 mg qw sc), body weight reduction from baseline and depletion ratio of fat to lean body mass were 15% and 1.5 in 68 weeks; estimated proportion of fat depletion in the visceral depot was 44% [82]. The remarkable ectopic/visceral fat depletion, which might be attributed to the anorexigenic and insulinotropic characteristics of these incretin-based medications (as discussed in *Metabolic surgery*), can lead to favorable metabolic consequences including improved hepatic insulin sensitivity/glycemic control and cardiovascular/renal outcomes. The *SCD2023* recommends that for patients with T2DM and established cardiovascular/renal disease or risks, a GLP-1RA should be considered as part of the treatment regimen [67]. The major CVOT also demonstrated that the GLP-1RA was associated with significant reductions in MACE (HR = 0.88), hospitalization for heart failure (HR = 0.91), and the composite kidney outcome (HR = 0.83) in patients with T2DM [87••].

### Dual Glucose-Dependent Insulinotropic Polypeptide (GIP)/GLP-1RA

As GLP-1, GIP is also a glucose-dependent insulinotropic hormone secreted from intestinal K cells and associated with favorable outcomes [76]. With the aim to boost the metabolic benefits of the incretins, by engineering GLP-1 activity into the GIP sequence, a dual RA, tirzepatide (with a unique pharmacological profile e.g., higher binding affinity for GIP than GLP-1 receptor) has been developed [88]. The SURPASS 1 to 5 trials indicated that (with a mean follow-up of 26 to 104 weeks) tirzepatide (5, 10, 15 mg qw sc) was associated with a significant reduction in HbA1c with MD ranging from − 1.69 to − 2.58% in patients with T2DM. Among these patients, 66.0 to 86.0% reached an HbA1c ≤ 6.5% [89•]. The weight reduction efficacy of tirzepatide seemed to be dose–response dependent as evidenced by 5%, 13%, 8%, and 13% of body weight reduction from baseline (BMI = 32.6 and 34.2 kg/m^2^) for 5 and 15 mg in 26 and 40 weeks; further follow-up suggested that the efficacy could be even stronger as a plateau in body weight was not reached with treatment durations shorter than 52 weeks [89•]. Body composition studies demonstrated that in patients with overweight or obesity (BMI = 37.9 kg/m^2^) receiving tirzepatide (5, 10, 15 mg), body weight reduction from baseline was 15%, 20%, 21% and mean depletion ratio (for all doses) of fat to lean body mass was 3 after 72 weeks [90]; in patients with T2DM with BMI ranging from 33.1 to 34.5 kg/m^2^, tirzepatide (5, 10, 15 mg) was associated with 23 to 38% and 21 to 29% of fat depletion in liver and visceral depots after 52 weeks [91]. Though results of the SURPASS CVOT (a dedicated cardiovascular outcomes trial for Tirzepatide) are due to report in October 2024, the pre-specified data from SURPASS 1 to 5 and J-mono suggest that tirzepatide (mean assigned dose: 9.9 mg qw) might potentially decrease the risks of MACE and all-cause mortality in T2DM patients (as evidenced by HR of 0.80, although this did not reach statistical significance) [92].

### Triple GIP/GLP-1/Glucagon RA

Glucagon is a peptide hormone secreted from pancreatic α-cells. It is a strong stimulator of hepatic glucose output [93]. In a phase 2 trial (with a mean follow-up of 24 weeks), the glucagon receptor antagonist LY2409021 (10, 20 mg qd sc) significantly reduced HbA1c (MD =  − 0.78%, − 0.92%) in patients with T2DM (compared with placebo). However, the glucose-lowering efficacy tailed off with higher doses (30 mg, 60 mg qd) [94]. This is consistent with the observation that the same compound (100 mg) given as a single dose, demonstrated no improvement in glucose tolerance in patients with T2DM [95]. Mechanistic studies further confirmed that though being glucotropic, glucagon is also a potent insulinotropic hormone with antidiabetic potential [96]. A triple GIP-GLP-1/glucagon RA with a GIP backbone and GLP-1/glucagon activities, retatrutide, has thus been developed to maximize the metabolic benefits [97]. With respect to the glucose-lowering efficacy, retatrutide (doses escalating from 2/3 mg to 12 mg qw sc) significantly reduced HbA1c (MD =  − 1.20%, − 2.02%, − 2.16% in 12, 24, 36 weeks) (vs. − 0.60%, − 1.41%, − 1.36% for dulaglutide (1.5 mg qw)) in patients with T2DM [98, 99•]. Retatrutide was also associated with 10% and 17% of reduction from baseline (BMI = 30.3, 36.0 kg/m^2^) in 12 and 36 weeks (vs. 0.04%, 2% for dulaglutide) [98, 99•]. 

### Combined GLP-1RA and Amylin Analog

Amylin is a peptide hormone co-secreted with insulin from pancreatic β-cells. With respect to glycemic control, amylin could delay gastric emptying, flatten postprandial glucose spikes, and thereby reduce HbA1c [100]. A meta-analysis of 4 RCT (with a mean follow-up of 16 to 52 weeks) showed that in addition to basal insulin, the amylin analog, pramlintide (120 to 150 μg bid/tid sc) significantly reduced HbA1c (MD =  − 0.33%) in patients with T2DM (compared with placebo or other antidiabetic medications) [101]. A recently published phase 2 RCT demonstrated that the amylin analog, cagrilintide (2.4 mg qw sc) was associated with HbA1c reduction (MD =  − 0.90%) in 32 weeks; there was a further reduction (MD =  − 1.30% with statistical significance) when given concurrently with semaglutide (2.4 mg qw sc) [102•]. 

The combined pramlintide and basal insulin could significantly reduce 2% of body weight from baseline (BMI = 33.9 kg/m^2^) in patients with T2DM [101]. Cagrilintide (2.4 mg) was associated with a significant reduction of 8% from baseline (BMI = 34.3 kg/m^2^); a further significant 8% of reduction was also observed in the combination of cagrilintide and semaglutide [102•].

## Future Development

To improve patient compliance, nonpeptide/small molecule GLP-1 RA in oral formulation has also been in development. A recently published phase 2 RCT (with a mean follow-up of 16 weeks) demonstrated that compared with placebo, danuglipron (129 mg bid po) remarkably reduced HbA1c (MD =  − 1.16%) and 4% of body weight from baseline (BMI = 33.3 kg/m^2^) in patients with T2DM [103•]. Moreover, orforglipron (doses escalating from 2 to 40 mg qd po) was associated with a significant 12.6% and 14.7% of body weight reduction from baseline (BMI = 37.8 kg/m^2^) at week 26 and week 36 in patients with overweight or obesity [104].

Other GLP-1-based medications, for instance, a recently developed GIP receptor antagonist conjugated with a GLP-1 peptide (with extended half-life) demonstrated superior metabolic benefits in animal studies and hence may become a therapeutic approach for T2DM and its complications in the future [105].

## Conclusions

Reduction of dietary glucose load can improve glycemic control in patients with T2DM. However, compared with metabolic surgery, an energy-restricted diet is associated with much less metabolic benefits due to their limited efficacy to reduce body fat. Given the strict eligibility criteria for metabolic surgery, newer pharmaceutical antidiabetic approaches, especially those incretin-based medications, with remarkable reduction effects on blood glucose as well as body fat (in ectopic/visceral depots), should be prioritized in treating T2DM as they have been proved metabolic beneficial in preventing/ameliorating cardiovascular and renal complications. In conclusion, to improve the metabolic outcomes of patients with T2DM, the Yin paradigm—body fat depletion must merge with the Yang paradigm—glucose-lowering treatments (Fig. 1).
